# Insight into the sialome of the castor bean tick, *Ixodes ricinus*

**DOI:** 10.1186/1471-2164-9-233

**Published:** 2008-05-20

**Authors:** Jindřich Chmelař, Jennifer M Anderson, Jianbing Mu, Ryan C Jochim, Jesus G Valenzuela, Jan Kopecký

**Affiliations:** 1 Faculty of Science, University of South Bohemia, České Budějovice, Czech Republic; 2 Vector Molecular Biology Unit, Laboratory of Malaria and Vector Research, National Institute of Allergy and Infectious Diseases, National Institutes of Health, Rockville, Maryland, USA; 3 Biology Centre of the Academy of Sciences of the Czech Republic, v.v.i., České Budějovice, Czech Republic

## Abstract

**Background:**

In recent years, there have been several sialome projects revealing transcripts expressed in the salivary glands of ticks, which are important vectors of several human diseases. Here, we focused on the sialome of the European vector of Lyme disease, *Ixodes ricinus*.

**Results:**

In the attempt to describe expressed genes and their dynamics throughout the feeding period, we constructed cDNA libraries from four different feeding stages of *Ixodes ricinus *females: unfed, 24 hours after attachment, four (partially fed) and seven days (fully engorged) after attachment. Approximately 600 randomly selected clones from each cDNA library were sequenced and analyzed. From a total 2304 sequenced clones, 1881 sequences forming 1274 clusters underwent subsequent functional analysis using customized bioinformatics software. Clusters were sorted according to their predicted function and quantitative comparison among the four libraries was made. We found several groups of over-expressed genes associated with feeding that posses a secretion signal and may be involved in tick attachment, feeding or evading the host immune system. Many transcripts clustered into families of related genes with stage-specific expression. Comparison to *Ixodes scapularis *and *I. pacificus *transcripts was made.

**Conclusion:**

In addition to a large number of homologues of the known transcripts, we obtained several novel predicted protein sequences. Our work contributes to the growing list of proteins associated with tick feeding and sheds more light on the dynamics of the gene expression during tick feeding. Additionally, our results corroborate previous evidence of gene duplication in the evolution of ticks.

## Background

Hard ticks (family Ixodidae) are well known ecto-parasites of vertebrates, with worldwide distribution and high medical importance due to their extraordinary ability to transmit various disease agents. Among hard ticks, the genus *Ixodes *is one of the most important vectors of human diseases. Lyme borreliosis, human granulocytic anaplasmosis (ehrlichiosis) and babesiosis are the main diseases transmitted by *I. scapularis *and *I. pacificus *in North America. In addition to these diseases, *Ixodes ricinus *in Europe and *Ixodes persulcatus *in Asia transmit tick-borne encephalitis (TBE). An increase of TBE-virus (Flaviviridae) prevalence among *I. ricinus *ticks in middle Europe has been recorded during the last few decades and the findings of *I. ricinus *at higher altitudes (above 1000 m) suggest that it is spreading to new areas, which correlates with the spread of human TBE cases [1]. Ticks and tick-borne diseases are becoming a more important health issue and detailed knowledge of the interactions among the tick, host and pathogen is crucial for understanding the mechanisms of pathogen transmission. The knowledge of saliva components is the basis for further understanding of these interactions.

Tick saliva is a powerful mixture of hundreds of different proteins and other pharmacologically active molecules. The effects of tick saliva or SGE (salivary gland extract) on the host are well described in several reviews [2-4]. Hard ticks require an array of molecules to evade the host haemostatic and immune systems and for successful completion of feeding, which usually lasts for 7–9 days for *I. ricinus*. Tick saliva is able to inhibit all three components of the host haemostatic system: blood coagulation, platelet aggregation and vasoconstriction [3,5]. The innate immune response is altered by the impaired activation of complement, resulting in a decrease in chemokine production and subsequent inhibition of inflammation. Acquired immunity is affected as well; *in vitro *experiments showed the inhibition of T-cell proliferation after incubation with *Ixodes ricinus *saliva [6]. Salivary gland extract alters the production of many cytokines by immunologically responsive cells leading to immunosuppression [7]. The changes in the expression profile of different cytokines indicate a polarization from Th1 toward the Th2 branch of the immune response, which could be a disease-determining factor for tick-borne pathogens [8]. During the late eighties, it was discovered that tick saliva is able to facilitate the transmission of viruses to the host and to another co-feeding tick [9]. This phenomenon was called saliva activated transmission (SAT) and has since been revealed for additional pathogens such as *Borrelia spp*. [10].

During the past decade, there has been great progress toward the description of particular molecules responsible for the host immunomodulation. It was shown that tick saliva contains various protease inhibitors with anticoagulant activity [11-13], as well as anti-inflammatory and immunosuppressive activity [14,15]. Anti-inflammatory activity is a result of several pharmacologically active molecules, including prostaglandins [16,17], apyrase [18] and histamine-binding proteins belonging to the lipocalin family [19,20]. Additionally, immunosuppressive protein Salp15 was identified from *Ixodes *scapularis [21]. Moreover, Salp15 was identified as saliva activated transmission (SAT) factor that facilitates the establishment of *Borrelia burgdorferi *in the mammalian host [22]. Among other proteins, tick saliva contains a metalloprotease with fibrinolytic activity, involved in extracellular matrix remodeling [23], a group of anti-complement proteins [24,25] and an IL-2-binding protein [26]. Many of these proteins were discovered using a high-throughput approach [27] that opened a vast new area for tick research and helped reveal a large number of novel proteins with completely unknown function, as well as many homologous proteins. From this point of view, tick saliva is a rich source of medical compounds. As an example, the immunosuppressive tick salivary protein Salp 15 was shown to suppress the development of experimental asthma in mice [28] and, as an immunosuppressor, Salp 15 is believed to become useful during allogenic transplantation [29].

This work augments the existing knowledge of tick sialomes. High-throughput studies of tick salivary glands have been completed for several ticks, such as American species *I. scapularis, I. pacificus *and *Dermacentor andersoni *[30-33] or tropical tick *Amblyomma variegatum *[34]. This is the first high-throughput work on the European tick *Ixodes ricinus*, which is the most important disease vector in Europe. In this study, four cDNA libraries from salivary glands were constructed and 576 EST were sequenced and analyzed per library, resulting in a total of 2304 EST. The four libraries cover the main phases of an adult female tick feeding period: 1) unfed tick, 2) early phase of feeding (24 hours after attachment), 3) middle phase of feeding (4 days after attachment) and 4) late phase of feeding-engorged tick (7 days after attachment).

Although many proteins that we identified were already known from *I. scapularis *and *I. pacificus*, several novel putative proteins were discovered. Moreover, analysis of mRNAs expressed at four points in the blood meal enables us to gain insight into the dynamics of tick feeding, and indirectly sheds light on the evolution of tick genes and genome composition.

## Results

### Characterization of data

**Figure 1:**
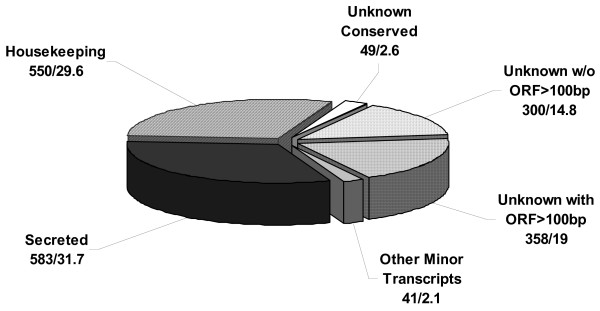
**Representation of the main transcript types**. Obtained ESTs were sorted into 6 main groups. Their representation in the obtained dataset is shown in Figure 1. Values represent EST number/% of EST.

Four different salivary gland cDNA libraries from *I. ricinus *females were analyzed in this work. The following feeding stages were used for mRNA isolation: Unfed (IRUF), 24 hours after attachment (IR24H), four days (IR4D) and seven days (IR7D) after attachment to the host. From the total of 2304 sequenced EST, 1881 were considered of high quality (less than 5% of undetermined base calls, phred quality ≥ 20, at least 80 bases not including poly A). The length of analyzed sequences (after removal of vector sequence) ranged between 84 and 1253 bp with average length of 503 bp. The sequences were clustered and aligned resulting in 1274 clusters comprising 268 contigs with two or more sequences and 1006 singletons. The high proportion of singletons is due to stringent analysis conditions rather than under-sampling; however, a larger dataset would be beneficial for statistic analysis. BLAST search was done for the consensus of each contig or singleton against the set of databases (NR, GO, KOG, CDD, Pfam, Smart, ACARI, rRNA and mitochondrial). The bioinformatic analysis was combined into a single Excel table where the clusters were manually annotated and sorted into functionally related groups based on the BLAST results from the various databases. Out of 1881 sequenced EST, approximately 30% (550) were identified as housekeeping genes due to a functional prediction and/or intracellular localization, and 32% (583) of ESTs contained a predicted signal for secretion from the cell suggesting their function in saliva (group of secreted genes). Several ESTs were 5' truncated, which restricted prediction by the SignalP server, yet had high similarity to secreted proteins on GenBank and were grouped with secreted genes. Forty-nine ESTs compiled in 37 clusters (3%) contained proteins that are conserved among different organisms but lack any functional prediction and were named 'unknown conserved'. The last largest group comprises 658 unknown transcripts (35%) without a predicted signal peptide and with no match to any database. There are three main reasons for such a high amount of unknowns and all of them probably participate in the resulting number: 1) Sequences contain only 5' untranslated region, 2) Truncated cDNA was cloned into the vector and only the part of 3' non-coding region was sequenced, and 3) Tick salivary glands contain many unknown and unique proteins with no similarity throughout all organisms. The group of unknowns was sorted for illustration between two groups, in accordance with the length of the open-reading frame; the threshold for the division was decided at the length 100 bp. Putative proteins similar to reverse transcriptases or transposases were classified as a single group. Although the protocol for cDNA library construction is designed for elimination of other than messenger-type of RNA, several ribosomal RNAs were sequenced, which is due to AT-rich profile of such transcripts. Main data characteristics are summarized and visualized in Figure 1.

### Distribution of transcripts among libraries

**Figure 2:**
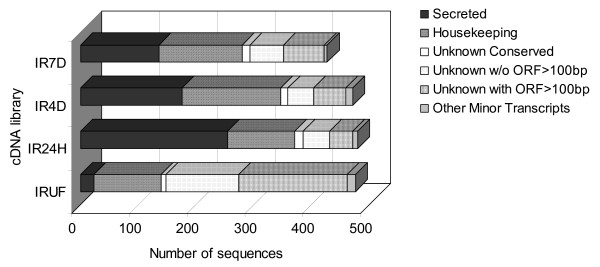
**Transcript distribution throughout each analyzed cDNA library**. IRUF – *Ixodes ricinus *unfed, IR24H – *I. ricinus *24 hours after attachment, IR4D – *I. ricinus *4 d after attachment, IR7D – *I. ricinus *7 d after attachment.

All libraries contain a similar number of high-quality sequences ranging from 432 for IR7D to 492 for IR4D. Figure 2 shows the distribution of the main groups among all libraries. The distribution of different gene types varies markedly. There is a large increase in the expression of secreted proteins between IRUF and IR24H libraries, while in IR4D and IR7D the ratio of secreted proteins decreases. This trend results from the distribution of the three most abundant families of secreted proteins: Collagen-Like Secreted Proteins (CLSP), Basic Tail Secreted Proteins (BTSP) and proteins containing Kunitz domains (Kunitz). These three groups represent only 1.7% of all transcripts in IRUF, but 37.6% in IR24H, 20.6% in IR4D and 17.1% in IR7D library. We can assume that during the early phase of feeding, the tick utilizes a large amount of energy for the production of proteins that enable it to feed successfully despite the host defense system. During the first few days after attachment, it is mainly innate defense that the tick has to fight against in a naïve host, and all three groups mentioned above could be involved in anti-haemostatic activity. During the later phases of feeding, the tick starts to digest and process the ingested meal while the site of attachment is already full of pharmacologically active molecules. The slight increase in production of housekeeping genes in the last two stages of feeding is in accordance with the need for higher metabolic activity during rapid growth of the salivary glands. However, at least two groups of secreted proteins appear to be produced in later phases of feeding. Histamine-binding proteins (HBP) and 4.9 kDa proteins are significantly overrepresented in the IR7D library (Table 3). The function of the 4.9 kDa group is completely unknown but HBP are known for their anti-inflammatory activity.

Quite interestingly, 66.8% (323 out of 483 EST) of the unknown proteins are present in the IRUF library. The reason could be the lack of data from unfed tick salivary glands as only cDNA libraries from unfed *Argas monolakensis *and *Amblyomma americanum *were published to date in the GenBank. The lack of a signal peptide in full-length clones suggests housekeeping function of these peptides. In order to identify proteins up-regulated by feeding, a comparison of the four libraries was conducted. Only 38 contigs out of 1274 contain five or more ESTs, the threshold for statistical evaluation (Table 1); therefore, we conducted statistical analysis on both individual contigs and whole classes of peptides. While housekeeping genes were sorted into functional categories, classification of secreted proteins was based on their primary structure similarity because exact function is not usually known. The distribution and statistic evaluation of both housekeeping and secreted proteins are shown in Table 2 (housekeeping) and Table 3 (secreted). Accession numbers for the GenBank EST database in the following sections always represent the largest clone of each mentioned contig. All additional sequences, assembled contigs and other data from the analysis can be downloaded [see Additional file 1].

**Table 1:** Contigs with more than 5 ESTs and their distribution among the four libraries

				**IRUF**			**IR24H**			**IR4D**			**IR7D**		
						*p*			*p*			*p*			*p*
**Contig**	**Comments**	**Class**	**EST**	**obs**	**exp**	**IRUF/IR24H**	**obs**	**exp**	**IR24H/IR4D**	**obs**	**exp**	**IR4D/IR7D**	**obs**	**exp**	**ALL**
83	cytochrome oxidase 3	h/meten	23	1	5.91	**0.004**	1	6.02	**0.001**	12	5.80	**0.002**	9	5.28	**0.000**
78	cytochrome oxidase subunit 1	h/meten	22	0	5.65	**0.004**	2	5.75	0.060	8	5.54	**0.001**	12	5.05	**0.000**
88	cytochrome c oxidase subunit II	h/meten	17	6	4.37	0.070	1	4.45	0.101	4	4.28	0.285	6	3.90	0.215
89	ATP synthase F0 subunit 6	h/meten	16	1	4.11	0.062	2	4.19	0.241	5	4.03	**0.021**	8	3.67	**0.031**
41	collagen-like secreted protein	s/CLSP	14	0	3.59	**0.000**	10	3.66	**0.000**	1	3.53	0.177	3	3.22	**0.001**
106	elongation factor-1alpha	h/ps	14	3	3.59	0.719	4	3.66	0.184	6	3.53	0.071	1	3.22	0.332
46	collagen-like secreted protein	s/CLSP	12	0	3.08	**0.000**	11	3.14	**0.000**	1	3.02	**0.043**	0	2.76	**0.000**
87	16S mitochondrial RNA	rRNA	12	3	3.08	0.519	2	3.14	0.192	5	3.02	0.221	2	2.76	0.588
117	Monolaris II group	s/Kunitz	11	0	2.82	**0.000**	11	2.88	**0.000**	0	2.77	**0.021**	0	2.53	**0.000**
49	collagen-like secreted protein	s/CLSP	11	0	2.82	**0.036**	5	2.88	0.208	3	2.77	0.743	3	2.53	0.210
116	putative 19 kDa secreted protein	s/19	10	0	2.57	**0.000**	10	2.62	**0.000**	0	2.52	**0.028**	0	2.30	**0.000**
66	basic tail secreted protein	s/BTSP	10	0	2.57	**0.029**	5	2.62	0.131	2	2.52	0.570	3	2.30	0.165
56	collagen-like secreted protein	s/CLSP	8	0	2.05	**0.042**	0	2.09	**0.000**	8	2.02	**0.000**	0	1.84	**0.000**
42	collagen-like secreted protein	s/CLSP	8	0	2.05	**0.042**	0	2.09	0.148	2	2.02	**0.002**	6	1.84	**0.003**
23	collagen-like secreted protein	s/CLSP	7	0	1.80	0.057	0	1.83	0.058	0	1.76	**0.000**	7	1.61	**0.000**
48	collagen-like secreted protein	s/CLSP	7	0	1.80	**0.000**	7	1.83	**0.000**	0	1.76	0.066	0	1.61	**0.000**
63	basic tail secreted protein	s/BTSP	7	0	1.80	**0.001**	6	1.83	**0.002**	1	1.76	0.164	0	1.61	**0.004**
68	basic tail secreted protein	s/BTSP	7	0	1.80	**0.001**	6	1.83	**0.002**	1	1.76	0.164	0	1.61	**0.004**
118	cytochrome b	h/meten	7	0	1.80	0.178	2	1.83	0.182	0	1.76	**0.003**	5	1.61	**0.013**
85	16S mitochondrial RNA	rRNA	7	1	1.80	0.294	3	1.83	0.204	3	1.76	0.116	0	1.61	0.310
130	60S ribosomal protein L22	h/psrp	7	3	1.80	0.365	2	1.83	0.182	0	1.76	0.173	2	1.61	0.442
14	collagen-like secreted protein	s/CLSP	6	0	1.54	0.078	0	1.57	**0.000**	6	1.51	**0.000**	0	1.38	**0.000**
134	60S ribosomal protein L3	h/psrp	6	6	1.54	**0.000**	0	1.57	0.079	0	1.51	0.089	0	1.38	**0.001**
13	collagen-like secreted protein	s/CLSP	6	0	1.54	**0.003**	5	1.57	**0.003**	0	1.51	0.204	1	1.38	**0.014**
5	collagen-like secreted protein	s/CLSP	6	0	1.54	0.078	0	1.57	0.189	2	1.51	**0.023**	4	1.38	**0.041**
133	40s ribosomal protein S27	h/psrp	6	4	1.54	**0.019**	0	1.57	0.189	2	1.51	0.215	0	1.38	0.070
159	5.3 kDa secreted protein	s/4.9	5	0	1.28	0.107	0	1.31	0.109	0	1.26	**0.000**	5	1.15	**0.001**
64	basic tail secreted protein	s/BTSP	5	0	1.28	**0.001**	5	1.31	**0.001**	0	1.26	0.121	0	1.15	**0.003**
7	collagen-like secreted protein	s/CLSP	5	0	1.28	**0.001**	5	1.31	**0.001**	0	1.26	0.121	0	1.15	**0.003**
137	NADH dehydrogenase 3	h/meten	5	0	1.28	0.244	1	1.31	0.248	0	1.26	**0.004**	4	1.15	**0.021**
24	collagen-like secreted protein	s/CLSP	5	0	1.28	0.244	1	1.31	**0.014**	4	1.26	**0.008**	0	1.15	**0.037**
158	basic tail secreted protein	s/BTSP	5	2	1.28	0.191	0	1.31	0.054	3	1.26	0.059	0	1.15	0.153
50	collagen-like secreted protein	s/CLSP	5	0	1.28	0.199	2	1.31	0.096	3	1.26	0.059	0	1.15	0.157
142	Monolaris II group	s/Kunitz	5	0	1.28	0.199	2	1.31	0.096	3	1.26	0.059	0	1.15	0.157
157	60S ribosomal protein L7A	h/psrp	5	3	1.28	0.058	0	1.31	0.187	2	1.26	0.208	0	1.15	0.158
154	60S ribosomal protein L31	h/psrp	5	3	1.28	0.124	1	1.31	0.248	0	1.26	0.258	1	1.15	0.301
151	40S ribosomal protein S12	h/psrp	5	2	1.28	0.492	1	1.31	0.476	2	1.26	0.208	0	1.15	0.560
131	unknown	uk	5	2	1.28	0.382	2	1.31	0.202	0	1.26	0.258	1	1.15	0.562

*P *values show significance of expression difference between two libraries; *P *– ALL shows which contig has random and non-random distribution. Significant values (*P *≤ 0.05) are marked in bold. Obs – observed, Exp – expected, h/– housekeeping, s/– secreted, 19 – 19 kDa protein, 4.9 – 4.9 kDa protein, BTSP – basic tail secreted protein, CLSP – collagen-like secreted protein, Kunitz – Kunitz domain containing protein, meten – energy metabolism, ps – proteosynthesis, psrp – proteosynthesis ribosomal protein, rRNA – ribosomal RNA, uk – unknown.

**Table 2:** Distribution of groups of housekeeping genes among the four libraries

			**IRUF**			**IR24H**			**IR4D**			**IR7D**		
						*p*			*p*			*p*			*p*
**Name of group**	**Contigs**	**EST**	**Obs**	**Exp**	**IRUF/IR24H**	**Obs**	**Exp**	**IR24/IR4D**	**Obs**	**Exp**	**IR4D/IR7D**	**Obs**	**Exp**	**ALL**
Metabolism energy	72	184	19	47.25	**0.000**	28	48.13	**0.000**	60	46.37	**0.000**	77	42.26	**0.000**
Protein synthesis – ribosomal proteins	82	166	49	42.63	0.293	46	43.42	0.240	49	41.83	**0.005**	22	38.12	**0.018**
Protein modification and degradation	32	36	5	9.24	0.069	13	9.42	0.222	8	9.07	0.484	10	8.27	0.275
Protein synthesis	11	27	8	6.93	0.381	5	7.06	0.252	9	6.80	0.332	5	6.20	0.630
Transcription mechanism	21	24	9	6.16	0.238	7	6.28	0.378	4	6.05	0.292	4	5.51	0.470
Signal transduction	20	20	8	5.14	**0.025**	1	5.23	0.058	6	5.04	0.640	5	4.59	0.151
Metabolism carbohydrates	14	17	1	4.37	**0.047**	2	4.45	**0.001**	11	4.28	**0.001**	3	3.90	**0.002**
Cytoskeleton related proteins	10	14	4	3.59	0.782	4	3.66	0.405	2	3.53	0.356	4	3.22	0.816
Intracellular trafficking mechanism	12	12	1	3.08	0.113	5	3.14	**0.045**	6	3.02	**0.017**	0	2.76	**0.041**
Nuclear structure related proteins	10	12	4	3.08	0.475	4	3.14	0.207	1	3.02	0.241	3	2.76	0.594
Metabolism	6	8	2	2.05	0.450	1	2.09	0.450	2	2.02	0.391	3	1.84	0.726
Metabolism – aminoacids	6	8	2	2.05	0.148	0	2.09	**0.044**	4	2.02	0.161	2	1.84	0.253
Metabolism – lipids	7	8	2	2.05	0.941	2	2.09	0.473	1	2.02	0.264	3	1.84	0.739
Cell Cycle	4	5	0	1.28	0.107	0	1.31	0.187	2	1.26	0.064	3	1.15	0.110
Metabolism – nucleic acids	5	5	1	1.28	0.242	0	1.31	0.054	3	1.26	0.120	1	1.15	0.284
Metabolism – ionts	4	4	1	1.03	0.306	0	1.05	**0.026**	3	1.01	**0.028**	0	0.92	0.116

*P *values show significance of expression difference between two libraries; *P *– ALL shows which contig has random and non-random distribution. Significant values are (*P *≤ 0.05) are marked in bold. Obs – observed, Exp – expected.

### Housekeeping genes

The group of housekeeping genes was divided among more detailed functional subgroups. The subgroups comprise a large number of diverse proteins with diverse intracellular functions (Table 2). As expected, proteins involved in energetic and nutrient metabolism, such as compounds of the respiratory chain, various ATPase subunits or enzymes participating in the metabolism of carbohydrates, amino acids and lipids, are well represented in the dataset, comprising 217 out of a total 1881 ESTs (11.5%). Their expression increases during feeding, probably due to higher metabolic requirements during digestion of the blood meal (Table 2). For the sake of simplification six proteins were compiled together in a group labeled metabolism. The group (metabolism) includes proteins with various metabolic roles. There are two proteins involved in defense and detoxification. Two ESTs coding for dopachrome-tautomerase, the key enzyme in the process of melanization [35] were detected in the IR4D library (Contig 259, EY199700). Another protein, possibly involved in detoxification, is a peptide similar to nicotinamide N-methyltransferase and is represented by only one EST in IR7D library (Contig 946, EY200443). This enzyme is engaged in N-methylation of nicotinamide and other pyridines to form pyridinium ions. Its activity is important for biotransformation of many drugs and xenobiotic compounds in humans and other mammals. Its function in ticks or other arthropods is not clear; moreover, there is no previous report of a nicotinamid N-methyltransferase in any arthropod species. Although the cDNA is truncated, the e-value (2.10^-5^) is convincing and BLASTp search detected a methyltransferase domain; thus we can assume that the 33% identity and 57% similarity between contig 946 and human nicotinamide N-methyltransferase (NP_006160) strongly suggest the homology between tick and human proteins. Other proteins with various metabolic activities included in this group are those involved in coenzyme transport and metabolism or synthesis of secondary metabolites (Contig 323, EY200796; 543, EY199519; 815, EY200185; and 825, EY200205).

The group of proteins involved in post-translational modification and protein degradation contains various heat-shock proteins, proteases, subunits of proteasome machinery and glycosyltransferases, among others. Proteosynthetic proteins involved in translation, including ribosomal proteins, represent 10.3% of the total ESTs (n = 193 EST). There is a significant decrease in expression of ribosomal proteins between IR4D and IR7D. This suggests that there is a decrease in the production of salivary proteins during the final phase before detachment, which is supported by the putative expression profile of secreted proteins (Figure 2).

The group of cytoskeletal proteins is represented by actin, actin-related proteins, myosin, dynein, alpha tubulin, collagen precursor and a protein similar to microtubule-binding protein called translationally-controlled tumor protein (TCTP). TCTP functions in chromosome partitioning during the cell division [36], but it also stimulates the release of histamine by basophils [37]. Homologues of TCTP were found in other ixodid ticks and are referred to as histamine release factors (HRF). One of them was detected in the saliva of *Dermacentor variabilis*, despite the lack of a putative signal peptide. In the same work recombinant tick TCTP/HRF proved its histamine releasing features [38]. The *I. ricinus *TCTP/HRF transcript is truncated at the 5' region, but its high homology with *I. scapularis *(e-value = 7e-86), which lacks a signal peptide, suggests that the signal peptide is not present in *I. ricinus *TCTP/HRF as well. TCTP/HRF seems to be expressed constitutively and independently during feeding [39]. In light of the fact that the only transcript found in our library originates from unfed ticks (Contig 999, EY200552) and that TCTP/HRF proteins are well conserved among different organisms, we can assume that the main function of tick TCTP/HRF homologue is a function of tick physiology although the presence of TCTP/HRF in *D. variabilis *saliva suggests some function in the host.

Proteins associated with the transport and metabolism of ions create a single group with four singletons. The group contains manganese superoxide dismutase (Contig 660, EY199845), CutA1 divalent ion tolerance protein homologue (Contig 744, EY200030), ferritin (Contig 590, EY199666) and protein similar to Rhodanese-related sulfur transferase (Contig 1131, EY200876). Superoxide dismutase catalyses the dismutation of superoxide into oxygen and hydrogen peroxide and functions as an important defense against superoxide radicals [40]. CutA1 divalent ion tolerance protein is found throughout all organisms and is involved in tolerance to divalent ions such as copper or iron ions. This is the first reported CutA1 from the ticks, where it may be important in tolerance to iron ions from the blood meal. Another protein connected to iron transport and deposition is ferritin, which is expressed in all tick tissues [41]. Ferritin is a cytosolic protein usually composed of 24 subunits and is involved in the storage of ferric ions. It was shown that *Ornithodoros moubata *ferritin is expressed constitutively and independently upon feeding in the midgut [42]. *Ixodes ricinus *salivary ferritin is represented by only one EST in IR4D library (Contig 590). Proteins involved in signal transduction and intracellular trafficking create two functionally related groups where proteins associated with G proteins, receptors, ion channels associated proteins and various transporters, among others, can be found. Contigs 294 (EY200035), 295 (EY200423), 299 (EY200084) and 802 (EY200163) were found in a group of cell cycle proteins and are most likely involved in the cell division cycle, however concrete functions remain unknown.

### Reverse transcriptase-like proteins

Besides housekeeping and secreted salivary proteins there are several transcripts similar to reverse transcriptases (RT) or reverse transcriptase-like proteins from other arthropods, mainly insects. It is interesting that 10 out of 11 ESTs similar to RT were found in the unfed tick library and only one originates from IR7D library, as though such mechanisms are suppressed during feeding. Additionally, eight ESTs are similar to RT only as reverse complement. The presence of anti-sense transcripts has been previously reported in eukaryotes and their regulative function on the transcription process has been proposed [43-45]. One clone (Contig 1006, EY200559) shows 51% similarity to a transposase from *Danio rerio *(CAK05416), and contig 255 (EY199575) displays 50% similarity to a mariner-like transposase (2124399A). The presence of these genes suggests an ongoing process of transposition in the tick genome. This mechanism could be responsible for the high duplication rate of some multigenic families. Another explanation is a lysogenic viral origin of these proteins.

### Putative secreted genes

The group of secreted genes includes many of the transcripts with predicted signal peptide and truncated transcripts with high similarity to secreted proteins from other tick species. Secreted proteins (both predicted and determined by BLAST) represent 32% of all ESTs. From the distribution among the libraries, it is obvious that the number of secreted protein transcripts is dramatically increased by feeding. There is an 11-fold increase in secreted molecules in the IR24H library compared to the IRUF library. During the last two stages of feeding the proportion of secreted proteins decreases while the number of housekeeping genes, mainly involved in energy and nutrient metabolism, is slightly higher (Figure 2). Table 3 shows the most abundant secreted groups with more than 5 ESTs and their distribution among the libraries.

**Table 3:** Distribution of the most abundant secreted genes (≥ 5 EST) among the four libraries

			**IRUF**			**IR24H**			**IR4D**			**IR7D**		
						*p*			*p*			*p*			*p*
**Name of group**	**Contigs**	**EST**	**obs**	**exp**	**IRUF/IR24H**	**obs**	**exp**	**IR24H/IR4D**	**obs**	**exp**	**IR4D/IR7D**	**obs**	**exp**	**ALL**
CLSP	71	209	2	53.67	**0.000**	104	54.67	**0.000**	62	52.67	0.102	41	48.00	**0.000**
BTSP	46	95	6	24.39	**0.000**	48	24.85	**0.000**	17	23.94	0.135	24	21.82	**0.000**
Kunitz domain	30	61	0	15.66	**0.000**	33	15.96	**0.000**	19	15.37	0.104	9	14.01	**0.000**
18.7 kDa	11	20	2	5.14	**0.001**	12	5.23	**0.003**	6	5.04	**0.029**	0	4.59	**0.001**
Metalloproteases	13	18	3	4.62	0.276	3	4.71	0.409	4	4.54	0.055	8	4.13	0.185
WC proteins	15	18	0	4.62	**0.031**	5	4.71	0.244	7	4.54	0.140	6	4.13	0.075
Ixodegrins	8	15	0	3.85	**0.000**	10	3.92	**0.002**	4	3.78	0.186	1	3.44	**0.002**
19 kDa	4	14	0	3.59	**0.000**	13	3.66	**0.000**	1	3.53	**0.025**	0	3.22	**0.000**
HBP	10	10	0	2.57	0.059	1	2.62	0.172	4	2.52	**0.044**	5	2.30	0.054
ISAC-like	7	7	0	1.80	0.178	2	1.83	0.348	3	1.76	0.327	2	1.61	0.431
Prokineticin domain	7	7	0	1.80	0.111	3	1.83	0.378	2	1.76	0.721	2	1.61	0.455
Salp15-like	7	7	0	1.80	**0.037**	4	1.83	0.089	1	1.76	0.514	2	1.61	0.194
6.78 kDa	4	6	0	1.54	0.198	2	1.57	0.208	3	1.51	0.210	1	1.38	0.360
4.9 kDa	1	5	0	1.28	0.107	0	1.31	0.109	0	1.26	**0.000**	5	1.15	**0.001**

*P *values shows significance of expression difference between two libraries; *P *– ALL shows which contig has random and non-random distribution. Significant values are (*P *≤ 0.05) are marked in bold. Obs – observed, Exp – expected.

#### Collagen-like secreted proteins (CLSP)

**Figure 3:**
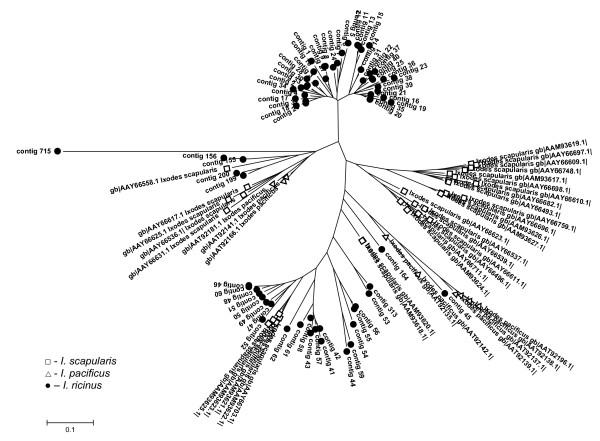
**Colagen-like secreted proteins (CLSP)**. Unrooted tree of CLSP family based on mature protein sequences and created by NJ algorithm. Only full-length sequences are included.

**Figure 4:**
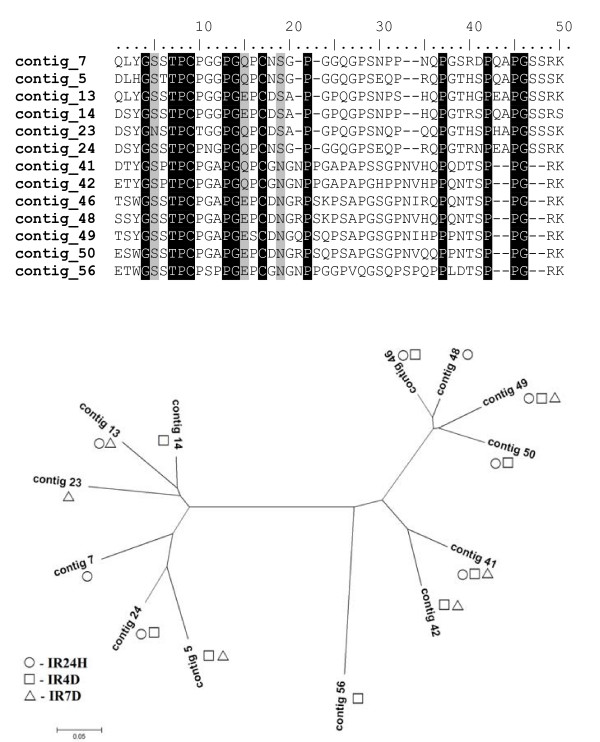
**Collagen-like secreted proteins with ≥ 5 EST; alignment and expression profile**. Alignment of CLSP contigs with more than 5 EST and graphical visualization of their representation in the libraries. Exact EST number of each contig is stated in Table 4.

The most abundant group in the combined library dataset comprises 209 ESTs within 71 contigs. Collagen-like secreted proteins are significantly upregulated by feeding, as only one contig with two ESTs was found in the IRUF library. The CLSP are small proline- and glycine-rich peptides with mature molecular mass ranging from 4.5 to 5.5 kDa in *I. ricinus*. In *I. pacificus *even smaller peptides (3.6 kDa) were found [31]. The name of the group refers to the high ratio of proline and glycine amino acid residues found in collagen. The presence of several X-Pro-Gly motifs points on the possibility of proline hydroxylation to hydroxyproline, which is responsible for collagen fibers production [46]. Hypothetically, CLSP may be able to create polypeptide chains at the site of attachment and function as the attachment glue or could affect the function of collagen in the interactions among cells, ligands and matrix [31,32]. Comparison of CLSP from *I. ricinus *with homologues from *I. scapularis *and *I. pacificus *reveals four major clades, one of which is exclusively found in *I. ricinus*, one is exclusively found in *I. scapularis *and the two other clades include transcripts from all three *Ixodes *species (Figure 3). The distribution of ESTs from this multigenic group among libraries implies the sequential expression of paralogs during the feeding process. Table 4 illustrates the temporal distribution of the CLSP clusters with more than 5 ESTs, their best match to the Acari database and distribution among the libraries. Some genes are expressed throughout all feeding stages after attachment while other genes appear to be expressed strictly within a specific feeding phase. There are two distinct major groups of CLSP that differ mainly in the length of C terminal region and create two distinct clades on the phylogram as shown in Figures 3 and 4. These groups likely originated from multiplication of two homologs. Others transcripts, scattered throughout the tree in Figure 3 diverged before the speciation of genus *Ixodes*. Alignment of CLSP contigs with ≥ 5 ESTs, their relationship and the library of contig origin are shown in Figure 4. The pattern of gene expression shows different timing for very similar peptides from the same clade. These proteins possibly play the same role in the tick or the host during different feeding periods. One possibility could be an antigenic shift in the most abundant proteins. *Ixodes ricinus *is a long term blood feeder; therefore, a specific immune response is raised against the tick by the host. The humoral branch of specific immune response is based on the elaboration of specific antibodies by B lymphocytes. The production of antibodies is activated by tick saliva that contains many different antigens. It is possible that antibodies raised against CLSP expressed during the early stages of feeding can be ineffective against CLSP that are produced later.

**Table 4:** The distribution of Collagen-Like Secreted Proteins (CLSP) with more than 5 EST among the libraries

**Contig**	**Best match to ACARI db [gb accession nr.]**	**e-value**	**EST**	**IRUF**	**IR24H**	**IR4D**	**IR7D**	***p***
**5**	CLSP11 [AAT92166.1]	3E-026	6	0	0	2	4	**0.041**
**7**	Putative secreted protein [AAY66696.1]	4E-024	5	0	5	0	0	**0.003**
**13**	CLSP11 [AAT92166.1]	8E-028	6	0	5	0	1	**0.014**
**14**	CLSP11 [AAT92166.1]	8E-028	6	0	0	6	0	**0.000**
**23**	CLSP11 [AAT92166.1]	9E-026	7	0	0	0	7	**0.000**
**24**	CLSP11 [AAT92166.1]	2E-027	5	0	1	4	0	**0.037**
**41**	Putative secreted protein [AAM93621.1]	3E-028	14	0	10	1	3	**0.001**
**42**	Putative secreted protein [AAM93621.1]	2E-029	8	0	0	2	6	**0.003**
**46**	Putative secreted protein [AAM93621.1]	2E-031	12	0	11	1	0	**0.000**
**48**	Putative secreted protein [AAM93621.1]	1E-031	7	0	7	0	0	**0.000**
**49**	Putative secreted protein [AAM93622.1]	2E-030	11	0	5	3	3	0.210
**50**	Putative secreted protein [AAM93622.1]	2E-031	5	0	2	3	0	0.157
**56**	CLSP1 [AAT92135.1]	1E-028	8	0	0	8	0	**0.000**

Difference in expression is represented by *P *value, significant difference is marked in bold.

Mechanisms of antigenic variability are well described for protozoan parasites and spirochetes [47], yet only one example has been reported for metazoan parasites thus far [48]. Antigenic variability is usually associated with an increased genome size and lower genome complexity, often due to recombination events [47]. This is the case of the parasitic protozoa in which the genome is much larger compared with their free-living relatives. Hard ticks possess almost twice as large genome as soft ticks and the complexity of hard tick genome appears to be much lower compared with soft ticks [49]. It is unknown if multigenicity and high recombination rates in hard ticks are related to antigenic variation as stated above or if it is the need for fast production of large amounts of immunoactive and other feeding-associated peptides. This question undoubtedly deserves further investigation.

#### Basic tail secreted proteins (BTSP)

**Figure 5:**
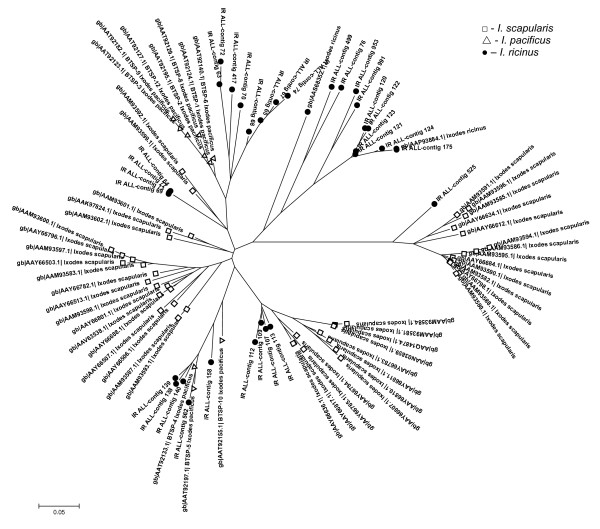
**Basic tail secreted proteins (BTSP)**. Unrooted tree of BTSP family based on protein sequences and created by NJ algorithm. Only full-length sequences are included.

Secreted proteins rich for lysine residues at the C terminus create the second most abundant peptide family with 95 ESTs in 46 contigs (5.1%). This group is well represented in both *I. scapularis *and *I. pacificus *salivary transcriptomes [31,32]. Anticoagulant Salp14 (AAY66785), which was found in *I. scapularis *and has been shown to inhibit factor Xa [13], is a member of the BTSP family suggesting an anticoagulant role for the whole family. The anticoagulation function of the BTSP family is also supported by the fact that positively-charged proteins can interfere with negatively-charged membranes of activated platelets [31]. The timing of protein expression of the BTSP related ESTs is similar to that of the CLSP family; up-regulation by feeding and then subsequent decrease between the IR24H and IR4D time points (Table 3). The phylogram in Figure 5 shows all BTSP sequences from the three *Ixodes *species. We can see a pattern similar to the CLSP group where each species creates its own clade. This suggests very fast gene duplication after speciation.

#### Peptides containing Kunitz domain

Kunitz-type domains are present mainly in inhibitors of trypsin and trypsin-like serine proteinases as chymotrypsin, kallikrein or plasmin. Another type of Kunitz-domain peptides lacking inhibitory properties are toxins from snake venom called dendrotoxins [50]. We found 31 clusters comprising 62 sequences of Kunitz domain-containing peptides expressed only in the stages after attachment. The expression of Kunitz proteins is highest during the first day after attachment (34 sequences) and then decreases throughout the next stages to 11 ESTs on the seventh day after attachment. Among the contigs containing Kunitz sequences, contig 117 was the most abundant with 11 ESTs found in the IR24H library.

**Figure 6:**
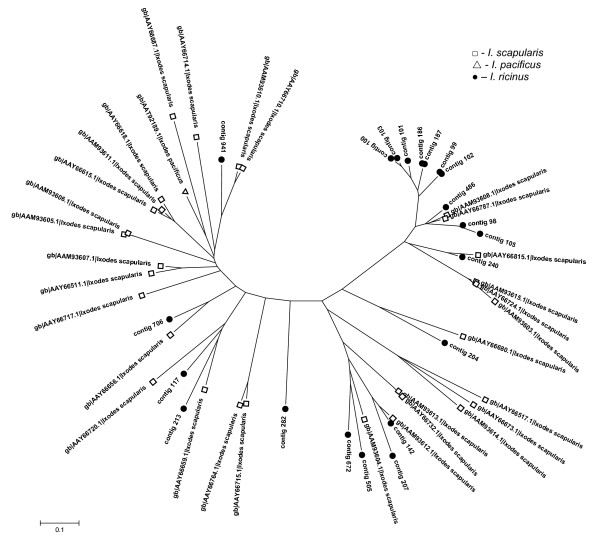
**Kunitz domain-containing peptides from monolaris II group**. Unrooted tree of all full-length monolaris II peptides from *Ixodes ricinus *and related sequences from *I. scapularis *and *I. pacificus*. The tree is based on protein sequences and created by NJ algorithm.

Twenty six contigs contain a single Kunitz-domain peptide with the general cysteine framework Xn-**C**-X8-**C**-X(16/18)-**C**-X5-**C**-X12-**C**-X3-**C**-Xn, similar or identical to monolaris II, according to nomenclature used by Francischetti et al. [31]. Within the monolaris II group, there are 11 very similar contigs containing a conserved SMGRL motif in the signal peptide cleavage site. An identical motif is also present in *I. scapularis *homologous group, suggesting common ancestral gene for both *I. ricinus *and *I. scapularis *groups. The phylogram in Figure 6 contains only full-length monolaris II sequences. Sequences from *I. ricinus *are scattered among sequences from *I. scapularis *and *I. pacificus *creating one large group (containing the SMGRL motif) that is common for *I. scapularis *and *I. ricinus*. The subgroup containing the SMGRL motif displays higher polymorphism than other Kunitz peptides. Contigs 771 (EY200101), 792 (EY200139) and 302 (EY200141) show similarity to tissue factor pathway inhibitor (TFPI) and contain Xn-**C**-X(7/9)-**C**-X15-**C**-X(5/7)-**C**-X12-**C**-X3-**C**-Xn framework, which resembles the monolaris III subgroup. Contig 400 contains two Kunitz domains and shows high similarity to *I. pacificus *Ixolaris-2 (AAT92212). One EST (contig 807, EY200173) is related to the five-domain (penthalaris) Kunitz proteins. Although it is not a full-length clone, similarity to penthalaris group is evident (81% identity with *I. scapularis *sequence, AAY66743; e-value = 4e-86). The rate of gene duplication appears to be slower in the Kunitz-domain groups compared with the BTSP and CLSP groups, in that each species chose two or three genes for rapid multiplication. We can presume that the Kunitz-domain group diverged before speciation of the genus *Ixodes*.

Kunitz domain-containing proteins function as inhibitors of serine proteases. Serine proteases can act as inhibitors of the blood clotting and coagulation systems. It has been shown that Ixolaris and Penthalaris inhibit the tissue factor pathway of blood clotting [11,12]. Monolaris subgroups can also play a role in anti-clotting and anti-coagulation activity, but such activity has not been proved thus far [32]. Another function could be deduced from the similarity with snake venom dendrotoxins that function as K^+ ^channel blockers [50].

#### 18.7 kDa group

**Figure 7:**
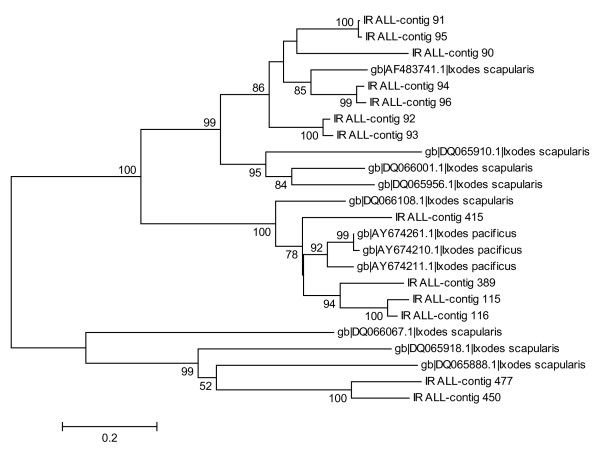
**18.7 kDa group of secreted proteins**. Cladogram of 18.7 protein group includes all *I. scapularis *and *I. pacificus *sequences obtained form the GenBank. Numbers represent bootstrap support of each clade with value above 50% (1000 rep.).

Fifteen clusters containing 34 ESTs showed similarity to an 18.7 kDa group reported previously from both *I. scapularis *and *I. pacificus *[31,32]. The overexpression of 18.7 kDa ESTs in the IR24H library (Table 3) suggests the role of this group during the early phase of feeding. The most abundant cluster (Contig116, EY200914) contains 10 ESTs from the IR24H library. The 15 clusters create three main clades together with *I. scapularis *and *I. pacificus *sequences (Figure 7). The mature peptides from all the three *Ixodes *species contain 12 conserved cysteines. Four of the *I. ricinus *contigs (Contig 115, EY199897; 116, EY199123; 389, EY199138; 415, EY199200) contain the same insertion with two additional cysteines as three *I. pacificus *and one *I. scapularis *sequences found on GenBank (for accession numbers see Figure 7). Contigs 115, 116 and 389 contain another insertion of six identical amino acids (YFDSHS), which appears to be unique for *I. ricinus *among all *Ixodes *species. Two contigs (Contig 450, EY199271; 477, EY199343) cluster together with three sequences from *I. scapularis *(for accession numbers see Figure 7) creating a single clade, which appears to have a common ancestral gene. The remaining sequences form the last and largest clade in Figure 5.

The 18.7 kDa group is comprised of at least three different polymorphic genes that share a conserved cysteine framework and few domains that suggest the same tertiary structure. The tertiary structure could be more important for protein function than the individual amino acid composition and conservation. Although we know nothing about the function of the 18.7 kDa family, the early production after attachment is typical for proteins used against blood coagulation and platelet aggregation.

#### Peptides containing Arg-Gly-Asp (RGD) domain

Integrins are cell receptors responsible for cell adhesion by recognition of an RGD motif on several extracellular matrix proteins such as fibrinogen, vitronectin, collagen or Von Willebrand factor [51]. RGD-containing proteins promote cell adhesion when insolubilized in the matrix, and inhibit cell-cell associations when they are soluble. Such soluble proteins are called disintegrins and were first reported from snake venom [52]. Tick peptides containing an RGD domain were shown to inhibit platelet aggregation by targeting GPIIbIIIa receptor and integrin αIIbβ3 [53-55]. Both *I. pacificus *and *I. scapularis *produce several types of RGD motif-containing proteins that are related to snake venoms [31,32]. Peptides with homology to disintegrins were also found in *I. ricinus*. Two non-homologous groups containing RGD motif were found and may act as disintegrins. The first group (Ixodegrins) is related to Ixodegrins described previously [31] and a snake venom component dendroaspin. Proteins in the second group are similar to prokineticin.

**Figure 8:**
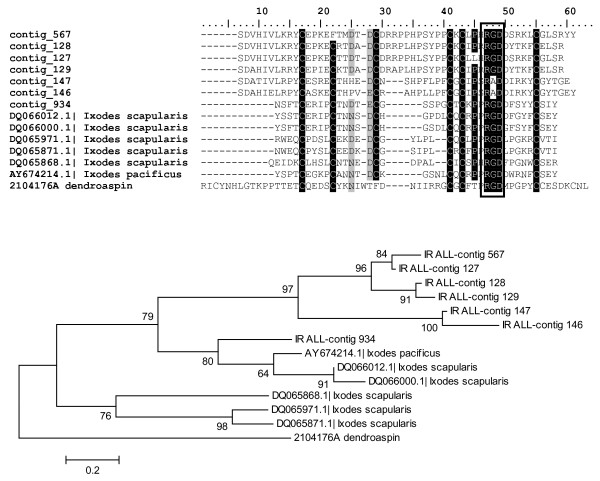
**Ixodegrins: Alignment and phylogram**. RGD motif, typical for disintegrins is marked in black rectangle. The tree was constructed by NJ method and numbers represent bootstrap support with value above 50% (1000 rep.). Dendroaspin isolated from *Dendroaspis jamesoni *was used as an outgroup.

The alignment in Figure 8 compares Ixodegrins from *I. ricinus, I. pacificus *and *I. scapularis*. Contig 934 (EY200415) shows high similarity to peptides found in other Ixodes ticks. The remaining *I. ricinus *contigs contain two insertions unique for *I. ricinus *and show high similarity to each other, suggesting another example of multigenic family. Contigs 146 (EY199187) and 147 (EY199165) contain an Arg-Ala-Asp (RAD) domain rather than a RGD. It was shown that a change from RGD to RAD can block the binding and thus disintegrin activity of the peptide [56]. Ixodegrins are strongly over-expressed 24 hours after attachment and then their expression decreases (Table 3).

**Figure 9:**
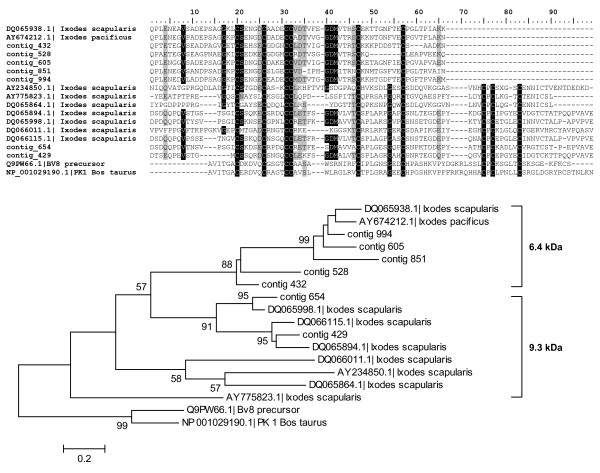
**Prokineticin domain-containing peptides**. Alignment and phylogram of both 6.4 and 9.3 kDa groups of prokineticin domain-containing proteins from the three Ixodes species. *Bombina variegata *protein Bv8 and prokineticin 1 from *Bos taurus *were used as an outgroup. The tree was constructed by NJ method and numbers represent bootstrap support with value above 50% (1000 rep.).

Peptides in the second group contain prokineticin (PK) domain and the group contains two subgroups related to Ixodegrin-2A (AAY66752) that differ in molecular mass. The alignment and phylogram of both subgroups is shown in Figure 9. The first subgroup includes acidic peptides with a mature molecular mass of 6.4 kDa and a conserved cysteine framework **C**-X5-**C**-X4-**C**-**C**-X(9–13)-**C**-X9-**C**. These peptides have no RGD motif; however, they contain one or even two XGD motifs in the loops between the cysteines. The second subgroup of PK domain-containing peptides includes acidic peptides with a molecular mass of 9.3 kDa and contain four additional cysteines at the C-terminus of 6.4 kDa subgroup creating the framework **C**-X5-**C**-X4-**C**-**C**-X(9–13)-**C**-X9-**C**-**X13-C-P-C-X(4–5)-C-X(4–7)-C**. The 9.3 kDa subgroup shows 36% identity and 50% similarity to astakin, a prokineticin domain-containing peptide isolated from *Pacifastacus leniusculus *(Q56R11) and 31% identity and 50% similarity to a prokineticin from *Bos taurus *(NP_001029190). Astakin was shown to be an important hematopoietic cytokine in invertebrates [57] and prokineticin can induce intestinal contraction in mammals [58]. Prokineticin domain proteins were originally identified as non-toxic peptides from black mamba (*Dendroaspis polylepis*) venom and later isolated from skin secretion of the frog *Bombina variegata *[59]. Prokineticin domain peptides appear to be involved in various biological processes such as control of circadian rhythm, differentiation of endothelial cells in steroidogenic glands and promotion of angiogenesis in endocrine tissues [60]. Although RGD motifs were found in *I. pacificus *and *I. scapularis *suggesting their disintegrin function, no RGD motif was found in *I. ricinus *sequences from the 9.3 kDa group. The lack of RGD implies a different function of the 9.3 kDa subgroup in *I. ricinus*. Prokineticin domain-containing peptides from both 6.4 and 9.3 kDa appear to be expressed equally during all three stages after attachment. The function of 6.4 kDa and 9.3 kDa subgroups remains to be tested.

#### Trp-Cys (WC) containing proteins

A group of proteins containing a Trp-Cys (WC) doublet has been found previously in the sialome of *I. scapularis *[32] and appeared to be unique for that species. We can now provide evidence that this group is common for at least *I. scapularis *and *I. ricinus*. Most of the WC-containing proteins share a similar seven-cysteine framework **C**-X11-**C**-X(13–15)-**C**-X3-**C**-X(10–14)-**C**-X(6–7)-**C**-X(22–26)-**C **and several very conserved residues that could be important for proper folding or activity of the mature protein. The most significant conserved residue other than cysteine is the tryptophan residue before the last cysteine residue. It has been proposed that the WC doublet can create a hydrogen bond resulting in a bend of the peptide chain. Fifteen contigs consisting of 18 ESTs with homology to WC containing proteins were identified in all cDNA libraries except IRUF. The expression of WC peptides is increased after attachment and the distribution of ESTs is equal among the three post-attachment libraries (Table 3). The function of this group remains unknown.

#### Histamine-binding proteins (HBP)

Ten genes coding for the proteins from the HBP lipocalin family were found in the IR24H, IR4D and IR7D libraries. The function and evolution of these proteins were described in detail elsewhere [20,61,62]. Briefly, tick lipocalins are able to bind histamine in a binding cavity resulting in the inhibition of inflammation, as histamine is a very potent mediator of the inflammatory response [20]. All *I. ricinus *sequences are similar to other *I. scapularis *and *I. pacificus *HBPs except contig 972, which shows only low similarity to other tick HBP (e-value = 5E-005). Additionally, BLASTp analysis found no HBP domain within the sequence, suggesting a potentially different function yet similar ancestral origin with other HBP coding genes. Histamine-binding proteins may only be expressed during the later phases of feeding as no sequences were captured in the unfed library and only one in IR24H.

#### Metalloproteases

Thirteen clusters consisting of 18 ESTs showed similarity to metalloproteases. All sequences were truncated; no full-length clones have been obtained. Contigs 218 (EY200183), 401 (EY199170) and 841 (EY200242) have extremely high identities (e-values = 1E-158, 1E-121 and 1E-97) with both *I. scapularis *and *I. pacificus *sequences, suggesting homology to coding genes. Notably, there is no feeding-induced associated overrepresentation of metalloproteases sequences (Table 3)

The main function of tick metalloproteases is probably related to anti-clotting activity at the site of attachment. Proteolytic metal-dependent activity toward fibrin(ogen), fibronectin and gelatin has been proved for *I. scapularis *saliva [23]; however, genes coding for metalloproteases have been found in 6 ixodid and 2 argasid species thus far, suggesting the presence of metalloproteases in saliva of more tick species.

#### Rare expressed genes similar to known proteins

##### α-2 macroglobulin

Alpha 2 macroglobulin is a protease inhibitor with a wide range of specificity [63]. The mechanism of inhibition is based on the capturing of the protease inside the large molecule of α-2 macroglobulin. This inhibitor has been detected in the hemolymph of *I. ricinus *and *Ornithodoros moubata *and possibly functions in tick innate immune defense against some pathogens [64]. Contig 680 contained one EST (EY199886) coding for a peptide with 34% identity and 55% similarity to a receptor region of α-2 macroglobulin previously found in *I. scapularis *(AAM93645) and *I. ricinus *(unpublished). The finding of another α-2 macroglobulin domain provides evidence that there are at least two different α-2 macroglobulin homologues in *I. ricinus*.

##### Calreticulin

Calreticulin is a protein highly conserved throughout all animals and usually functions in calcium storage. We have found only one clone in the IR4D library (Contig 614, EY199726) with 96% identity to *I. scapularis *calreticulin (AY271305) at the nucleotide level. It has been proposed that tick calreticulin is secreted into the host and possesses an immunoactive function [65].

##### Defensins

Defensins are small antimicrobial peptides common for both vertebrates and invertebrates. Defensins are important in defense against various microbial pathogens including *Borrelia spp*. [66]. We found two different defensins in the IR7D library (Contig 921, EY200392 and 969, EY200491). Contig 921 is a homologue of preprodefensin 1 and 2 isolated from *I. ricinus *(ABC88432, AAP94724) suggesting polymorphism in this gene. Contig 969 shows the highest similarity to Varisin A1 from *D. variabilis *(AAO24323).

##### Neuropeptide-like protein (NPL) with GYG repeats

Peptides with GYG repeats were found in *Caenorhabditis elegans *and show high antimicrobial activity against certain microbial organisms [67]. Three homologues (Contig 279, 494, 935) of NPL proteins from *I. pacificus *(AAT92111, AAT92131) have been identified in IR24H, IR4D and IR7D libraries. NPL proteins could act as antimicrobial peptides at the site of attachment [30] or they may be secreted into the hemolymph and be involved in tick humoral antimicrobial defense.

##### Carboxypeptidase inhibitor precursor

Contig 929 (EY200408) found in IR7D library showed 66% similarity (e-value = 1E-22) to a carboxypeptidase inhibitor from *Rhipicephalus bursa *salivary glands (AAW72225). The *R. bursa *carboxypeptidase inhibitor is a potent anticoagulant which accelerates fibrinolysis in blood clots [68]. This putative *I. ricinus *carboxypeptidase inhibitor adds to the growing family of tick anticoagulants.

##### Ixoderin B

Ixoderins are tick lectins related to ficolins, which are responsible for complement activation, and to arthropod lectins, which act as plasma agglutination activators. In tick plasma, lectins also play a role in antimicrobial activity, potentially against transmitted pathogens [69]. There are at least two ixoderin families in *I. ricinus*: Ixoderin A, which is expressed in all tick tissues and Ixoderin B expressed only in salivary glands [70]. Contig 617 (EY199735) is homologous to Ixoderin B (AAV41827) which implies the multigenicity of ixoderin B group. As ixoderins are related to lectins, they can be involved in tick innate immunity, but specific expression of Ixoderin B in salivary glands suggests also some immunomodulatory function in the host.

##### Keratinocyte associated protein 2-like protein (KAP2-like)

One transcript (Contig 624, EY199752) coding for KAP2-like protein was found in the IR4D library. KAP2-like proteins have been identified in almost all major groups of eukaryotes according to a tBLASTn search against the EST database of all organisms. Nothing is known about the function of KAP-2 protein, but it probably possesses important housekeeping functions as it is highly conserved among phylogenetically distant organisms.

##### Phospholipase A2 (PA2)

PA2 activity was found in *Ammblyoma americanum *saliva [71] and the genes homologous to the secreted type of PA2 were identified in other ixodid ticks, according to tBLASTn search against EST database. This suggests that PA2 activity in saliva is common for hard ticks. One clone (Contig 1024, EY200582) was found in the IRUF library and showed approximately 40% identity and 60% similarity to PA2 from various organisms.

##### Pitituary tumor transforming protein 1 interacting protein – like

Contig 982 (EY200521) displayed high similarity (45% identity, 64% similarity, e-value = 2E-30) to PTTG-1-IP from *Gallus gallus *(XP_422649). PTTG-1-IP is a protein that binds PTTG, a protein with multiple regulative functions in mitosis, gene expression, cell transformation and also angiogenesis [72]. This is the first report of PTTG-1-IP from invertebrates. The exact function of PTTG and PTTG-1-IP is not clear either for mammals or invertebrates.

#### Other minor secreted proteins

Many tick salivary proteins with a predicted signal peptide are novel peptides with no similarity among other organisms other than ticks or they are unique for *I. ricinus*. These proteins are named after the mature predicted molecular mass [see Additional file 1]. Many peptides are very small with molecular weight below 5 or 3 kDa. This may be due to sequencing artifacts creating false stop codons; however, small peptides can be functional in ticks. For example, there is one thrombin inhibitor isolated from the hard tick *Boophilus microplus *called microphilin with a molecular mass of about 2 kDa [73].

## Conclusion

The work presented here highlights several major themes concerning tick salivary proteins. There is an enormous overrepresentation of secreted protein transcripts after attachment and a subsequent shift to the production of molecules associated with energetic metabolism. This is probably a result of increased metabolic rate associated with blood ingestion and digestion. The difference in the production of mRNAs coding for secreted proteins between unfed ticks and ticks 24 hours after attachment is remarkable. An increase of 11 fold shows that hard ticks possess mechanisms allowing a rapid switch in physiology. While many secreted proteins appear to be induced after attachment, they are probably important in the feeding process. Protein production is an energetically demanding process so these proteins should have some function either in ticks or in the host. Similar abundance of secreted proteins among the three *Ixodes *species gives us an idea of which proteins could be crucial for successful feeding. The most abundant protein groups (i.e., CLSP, BTSP, Kunitz-domain or 18.7 kDa groups) are common for all three species and should be the first to be tested for any activity in the host. On the other hand, some proteins that have been tested for activity by other research groups do not belong into any of the most abundant groups. For example homologs of Salp15, the only salivary protein proven to be a SAT factor (peptide facilitating pathogen transmission) were found in only seven ESTs. The same EST number was found for the anti-complement proteins related to ISAC and IRAC isolated from *I. scapularis *and *I. ricinus*. Therefore, rare transcripts should also be evaluated for functionality. Hypothetically, abundant and strongly feeding-induced peptides can be aimed against innate defense mechanisms already active in the early phase of feeding. The less abundant proteins could possess more specific immunomodulatory function and could also participate in the SAT effect.

The most abundant protein families display interesting expression profiles throughout the feeding period and show temporal control of expression of very similar molecules. Feeding phase-specific expression, as was shown in the CLSP family, is an interesting phenomenon with several possible explanations: 1) It can be a result of gene arrangement on the chromosome without any further importance; 2) There can be sequential production of functionally identical peptides with different antigenicity and finally; 3) Similar peptides can have different functions in different stages of feeding. The second explanation is very tempting, because it would be a completely new aspect of the tick-host interaction, which is common among parasitic microorganisms, but very rare among metazoan. However, some points will need to be elucidated. We must know first whether the candidate proteins for possible antigenic variation are antigenic. If these molecules are antigenic they would then need to be checked for cross-reactivity by antibody recognition.

As there is a wide range of *I. ricinus *hosts, we can discuss the possibility of expression profile differences among ticks that were fed on different hosts. It was shown that different feeding conditions, including different hosts, result in protein profile changes of the saliva [74]. Ticks used in our work were fed on guinea-pigs, which are not natural host of *I. ricinus*; however, it is widely used laboratory model in tick research. Although there is no direct evidence of differences between sialomes of ticks fed on different animals, we assume that the data obtained with ticks fed on guinea-pigs can be applied to other hosts. Future research may be needed to test the possible effects of host influences on the tick sialome.

There is a high ratio of unknown transcripts in unfed ticks. To date, only one salivary gland library from unfed Ixodid ticks (*A. americanum*) was published in the GenBank. The lack of sequences from comparable feeding stage could be the reason for such high proportion of unknown transcripts in unfed library. As the main function of tick salivary glands during the starving phase is osmoregulation [75], some of unknown proteins could be involved in the process of water balance regulation.

The presence of multigenic, highly polymorphic families supports the theory of gene conversion and duplication in tick genome evolution. Comparison of the three phylogenetically close species shows an unusually high rate of gene duplication in many multigenic families. The cause of such fast gene multiplication in hard ticks is still not known; although, there is suspicion for the role of transposition in this process. It is notable that a number of organisms that use multigenic proteins, mainly for antigenic variation, are pathogens transmitted by hard ticks. It would be worthwhile to know whether there is any relationship between tick and transmitted pathogen multigenicity.

There are several abundantly secreted and blood meal-associated proteins that are probably very important for successful feeding. All of the most abundant proteins appear to be multigenic with an amount ranging from several to tens of paralogs. There is also a high probability of polymorphism and the number of homologs is probably higher than the number found in this work. Now, we have large databases of putative proteins expressed in tick salivary glands, but for the most part, the proper function is unknown. For some proteins a prediction based on sequence homology can be made; however, it is still unclear whether similar proteins from one family possess the same function as the homologs. On the whole, there are many salivary proteins that need to be tested for their function. It appears that a high-throughput approach would be highly beneficial for functional screening of tick salivary proteins. Additionally, the contribution of new sequences into the public databases will bring benefits for all the scientific community interested in tick and vector research or comparative phylogenetic studies.

As we unfold more peculiarities about tick genetics, thanks to the sialome research, we can see that ticks are interesting – not only for study of parasite-host interaction or searching for novel pharmacoactive molecules, but they prove to be an important model organism for disclosing some mechanisms of gene and genome evolution.

## Methods

### Ticks and tissue isolation

*Ixodes ricinus *adult females were obtained from the colonies maintained at the Institute of Parasitology of Czech Academy of Sciences in Ceske Budejovice. Pathogen-free ticks were fed on the guinea pigs complying with Act No. 207/2004 Coll. and approval AVCR 51/2005 given by the committee of Czech Academy of Sciences. Approximately 10 pairs of salivary glands were dissected from both unfed and fed adult ticks. Dissections were done in phosphate buffered saline (PBS); glands from each stage were washed in sterile ice-cold PBS, pooled together into a single tube and stored in RNAlater (Ambion, USA) until mRNA isolation.

### Synthesis of cDNA libraries and sequencing

Messenger RNA was isolated using Micro Fast Track mRNA isolation kit (Invitrogen, USA) according to the manufacturer protocol. Precipitated, washed and dried mRNA was diluted into 4 μl of DEPC-treated H_2_O and 3 μl of mRNA were used for first strand cDNA synthesis. The construction of cDNA libraries was done using the SMART cDNA library construction kit (BD Clontech, USA) according to the protocol provided by manufacturer, with some modifications. In order to determine optimal number of cycles, two identical amplification reactions were prepared. After 10^th ^amplification cycle the first one was stored on the ice, while the second one was used for the PCR cycles number optimization by removing 3 μl samples from the reaction every two cycles until cycle number 22. The samples were checked by visualization on an agarose gel. The optimal number of cycles with visible and equally represented products was used for the first amplification reaction (e.g. When 18 cycles were optimal, 8 additional cycles were used). The amplified DNA was treated with proteinase K, which was subsequently washed away by several washings with ultrapure water using Microcon YM-100 (Millipore). After Sfi I digestion and fractionation using a Chroma Spin-400 column (BD Clontech, USA) the fractions were checked using agarose gel and pooled into three tubes in a size-dependent manner (large, medium and small PCR products). Each pooled cDNA was washed with ultrapure water and concentrated to 4–7 μl using Microcon YM-100 column (Millipore). Three microliters from each tube were used for the ligation into the λTripleEx2 vector. The ligation reaction was packed into the phages using the Gigapack III Gold Packaging extract (Stratagene). Three libraries (large, medium, small) were constructed for each feeding phase resulting in total of 12 libraries. Each un-amplified library was plated onto LB agar plates aiming for approximately 300 clones per one 150 mm plate. Randomly selected clones were picked into the 96-well plate with 75 μl of water per well. Two plates were picked per library resulting in 6 plates for each feeding phase and 24 plates (2304 clones) for the experiment. Polymerase chain reaction with vector-specific primers (PT2F1 5'- AAGTACTCTAGCAATTGTGAGC -3' and PT2R1 5'- CTCTTCGCTATTACGCCAGCTG - 3') was done on selected clones. Cleaned PCR products were used as template for cycle-sequencing reaction using BigDye Terminator v3.1 cycle sequencing kit (Applied Biosystems, Foster City, CA) and forward primer PT2F3 primer 5' - CTCGGGAAGCGCGCCATTGT - 3'. Samples were directly sequenced on an ABI 96 capillary DNA sequencer (Applied Biosystems), or stored at -80°C.

### Bioinformatics

A detailed description of the bioinformatic treatment of the data appears elsewhere [33,76]. For this analysis, EST trace files were analyzed using a customized program based on the Phred algorithm [77]. Resulting sequences with average quality score greater than 20 were retained for subsequent analysis. Primer and vector sequences were removed from the 5' and 3' ends of the ESTs. The resultant sequences were grouped into clusters using a customized program (Cluster 6) based on identity (95% identity, 64 word size) and aligned into contiguous sequences (contigs) using the CAP3 sequence assembly program [78]. BLAST searches of individual contigs and singletons using executable programs obtained from the NCBI FTP site as previously described [76,79] were conducted against the non-redundant (NR) protein database of the NCBI, the gene ontology (GO) fasta subset [43], the conserved domains database (CDD) of NCBI [80] which contains the KOG [81], Pfam [82] and Smart databases [83] and to custom-downloaded databases containing ACARI (a subset containing mite and tick sequences), mitochondrial and rRNA nucleotide sequences available from NCBI. Peptides were submitted to the SignalP server as previously described [76,84] to detect signal peptides indicative of secretion. The individual cDNA libraries were directly compared with each other using a customized program (Count Libraries) that assesses the individual contribution of each individual library to the combined contig of interest. This analysis indicates putative proteins that may be over- or under-represented at a given time point. Chi-square analysis was conducted on contigs that contained more than 5 ESTs.

## Authors' contributions

JC carried out the conception and design of the work, tissue preparation, RNA isolation, cDNA libraries construction, preparation of samples for sequencing, obtained data interpretation and manuscript preparation. JMA carried out bioinformatic treatment of raw sequence data and participated in manuscript revision. JM participated in sequencing the libraries. RCJ participated in statistical evaluation of data and manuscript revision. JGV participated in the design of the work, manuscript revision and final approval of the version and JK carried out the conception and design of the work, participated in manuscript revision and approval of the final version.

## Supplementary Material

Additional file 1 Supplemental table. Zip file contains MS Office Excel table with links to text files, containing additional information on all ESTs. Click here for file
